# *Ceropegia jinshaensis* (Apocynaceae), a new species from northwestern Yunnan, China

**DOI:** 10.3897/phytokeys.130.34311

**Published:** 2019-08-29

**Authors:** Zhi-Kun Wu, Jie Cai, Lei Cai, De-Tuan Liu

**Affiliations:** 1 Department of Pharmacy, Guizhou University of Traditional Chinese Medicine, Guiyang, 550025, Guizhou, China; 2 Germplasm Bank of Wild Species, Kunming Institute of Botany, Chinese Academy of Sciences, Kunming 650201, Yunnan, China; 3 Yunnan Key Laboratory for Integrative Conservation of Plant Species with Extremely Small Populations, Kunming Institute of Botany, Chinese Academy of Sciences, Kunming 650201, Yunnan, China

**Keywords:** Apocynaceae, *
Ceropegia
*, new species, Yunnan, Yangtze River

## Abstract

*Ceropegia jinshaensis* D.T.Liu & Z.K.Wu (Asclepiadoideae, Apocynaceae), a new species from northwestern Yunnan along the upper Yangtze river of China, is described and illustrated. This species is similar to *C. meleagris* H. Huber, *C. dorjei* C. E. C. Fischer and *C. aridicola* W. W. Smith, but can be distinguished easily by its leaf shape and floral features, especially the corolla shape and size, the interior of corolla tube and coronal characters.

## Introduction

Traditionally, the genus *Ceropegia* Linn. (Apocynaceae, Asclepiadoideae) comprises more than 220 species which mainly occur in seasonally dry places in and around the semi-arid regions of the Old World, from China to the northern tip of Australia, India, Arabian Peninsula and southern Africa (Albers and Meve 2002, Surveswaran et al. 2009, Kambale et al. 2012, Bruyns et al. 2017). A recent phylogenetic study of *Ceropegia* with a far wider selection of species and more gene regions than before showed that the highly succulent stapeliads and *Brachystelma* R.Br. ex Sims are nested within *Ceropegia*. A revised and expanded classification of *Ceropegia* that included *Brachystelma* and all genera of the stapeliads was proposed with more than 700 species in 63 sections, making it by far the largest genus in the Apocynaceae (Bruyns et al. 2017).

According to the Flora of China, a total of 17 species of *Ceropegia* and 2 species of *Brachystelma* occur in China (Li et al. 1995), mainly distributed at low elevations in southwestern China and southern China.

During botanical exploration in northwestern Yunnan in 2015 and in 2018, an unknown species of Apocynaceae was collected on an open stony slope of the valley of the Jinsha River (upper part of Yangtze River). Its clear sap, tubular flower with a basal inflation, erect lobes coherent at their apices and forming a canopy and corona in two series, fit the main characters of *Ceropegia* in the narrow sense. After investigation of the specimens of *Ceropegia* from the main herbaria (PE, KUN and IBSC) in China, and careful consultation of the literature, especially newly published species from Asia (Swarupanandan and Mangaly 1992, Li et al. 1995, Albers and Meve 2002, Bhaskar 2006, Meve 2009, Kullayiswamy et al. 2013, Kidyoo 2014, 2015, 2018a, 2018b, Kidyoo and Paliyavuth 2016, 2017, Rodda and Meve 2017, Kumar et al. 2018), we concluded that this plant represents a species new to science and we describe it here.

## Materials and methods

Morphological observations and measurements of the new species were carried out based on living plants and dry specimens with Vernier calipers. Literature studies included relevant monographs and recently published papers of *Ceropegia* (see introduction). Specimens at PE, KUN and IBSC were checked via Chinese Virtual Herbarium (CVH, http://www.cvh.ac.cn) and KUN (http://kun.kingdonia.org), the images of type specimens of *C. meleagris* H. Huber and *C. dorjei* C. E. C. Fischer were obtained from JSTOR Global Plants (http://plants.jstor.org/).

## Results

### Taxonomic treatment

#### Ceropegia jinshaensis

Taxon classificationPlantaeGentianalesApocynaceae

D.T.Liu & Z.K.Wu sp. nov.

A481BAA8172750D983A32FFEA5C2C617

urn:lsid:ipni.org:names:77201383-1

Figs 1 , 2A–F

##### Diagnosis.

This species differs from the Nepalese *C. meleagris* by having heart-shaped leaves, a small and narrowed upper part of corolla tube, corolla lobes coherent at the top to form a pentagonal canopy and glabrous internal surface of corolla. It also differs from *C. dorjei* by having smaller corolla and differs from *C. aridicola* W. W. Smith by having a different shape of leaf apex, smaller flower, different corolla color and canopy structure.

##### Type.

CHINA. Yunnan: Ninglang county, in the vicinity of Ahai Dam, 27°19'56.80"N, 100°30'29.39"E, 1500 m a.s.l., on stony slope of dry and hot valley, 28 Aug 2015, *Z.K. Wu et al. LJBG2015130* (holotype KUN!, isotype KUN!).

##### Description.

Perennial herbs 25–35 cm tall; *Rootstock* consisting of many fusiform roots, 3–7 cm long, 0.2–0.4 cm diameter. *Stems* creeping or decumbent, not twining, up to 95 cm long, solitary or branched from base, pubescent. *Leaves* opposite, blade heart-shaped, 1.6–2.3 × 1.3–1.8 cm, both surfaces pubescent, somewhat fleshy, base cordate, apex acute to subacute, margin entire, petioles 1.4–2.3 cm, pubescent. *Inflorescences*, 1 to 3-flowered, on short peduncle, 4 mm long, pedicel 3–5 mm long, green or pinkish white, pubescent. *Sepals* deeply 5-parted, lobes ovate-lanceolate, greenish or reddish at tip, 3 × 1 mm, outside sparsely pubescent. *Corolla* tubular with lobes facing upwards and fused at tips, 8–12 mm long, outside pale-green towards mouth of tube with dark purple speckles at the top part, sparsely pubescent, inside white to pale green with dark maroon irregular swellings in basal inflation of tube towards mouth; *tube* 10–11 mm long, with basal inflation 6–8 mm long, 5.0–6.5 mm diameter, then abruptly narrowing to 2 mm diameter and remaining the same width at mouth; *lobes* 2–3 mm long, 1–2 mm broad at base, brown or purple both sides, joined at tips to form flat pentagonal canopy, apex emarginate or 2-cleft. *Corona* 4.0–4.5 × 4–5 mm, sessile; *outer lobes* 3–4 mm, notched in middle to ca. 1/3, pinkish with purple verrucae and many white hairs; *inner lobes* 2.5–3.0 mm, purple or pink, glabrous. *Follicles*, 3.8–5.5 cm long, 3.5–4.5 mm diameter in the middle, glabrous.

**Figure 1.**
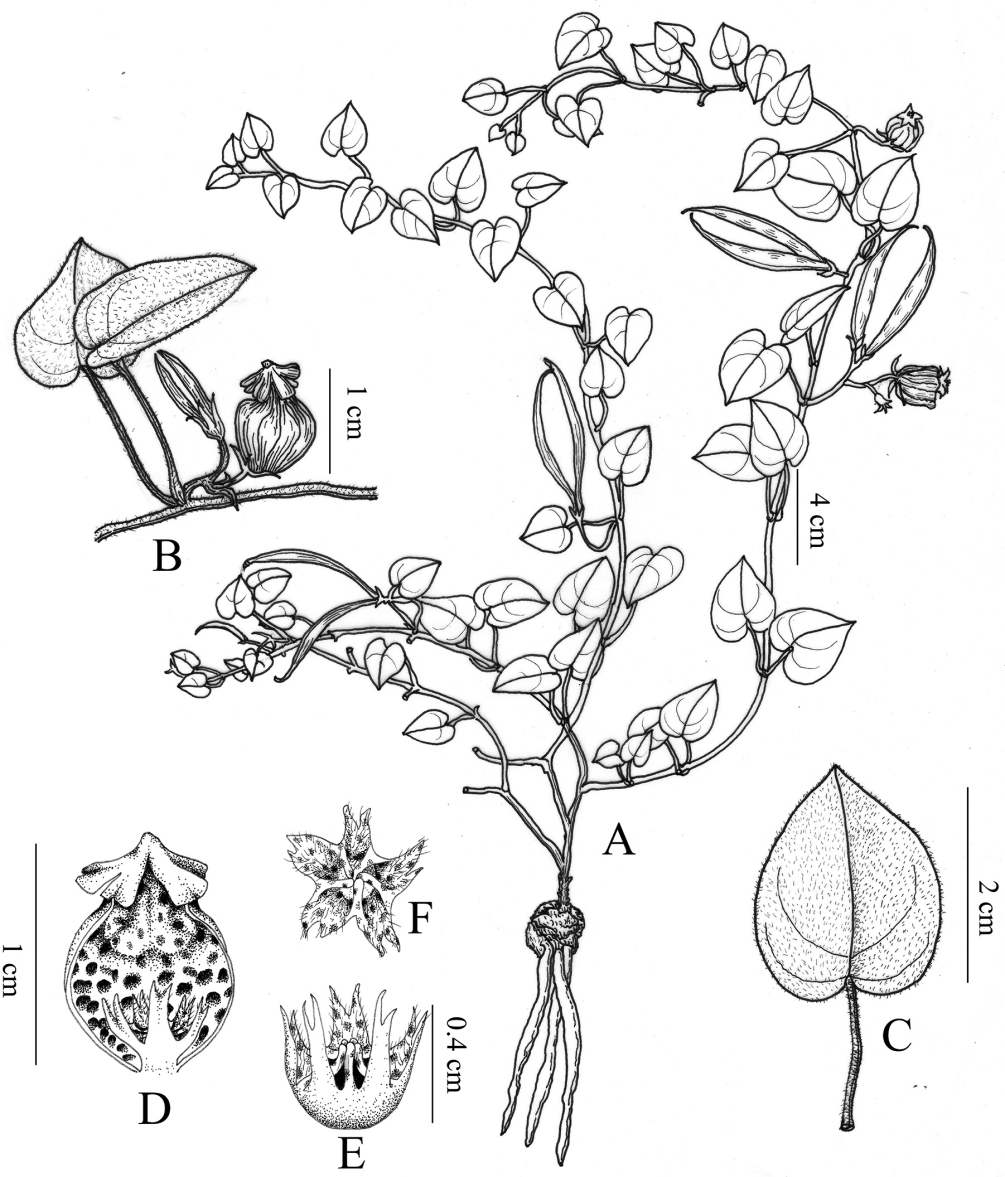
*Ceropegia jinshaensis* sp. nov. **A** plant **B** flower with young follicles **C** leaf **D** corolla tube dissected showing corolla interior and corona position **E** side view of corona **F** front view of corona. Drawn by Rongmei Zhang from holotype.

**Figure 2.**
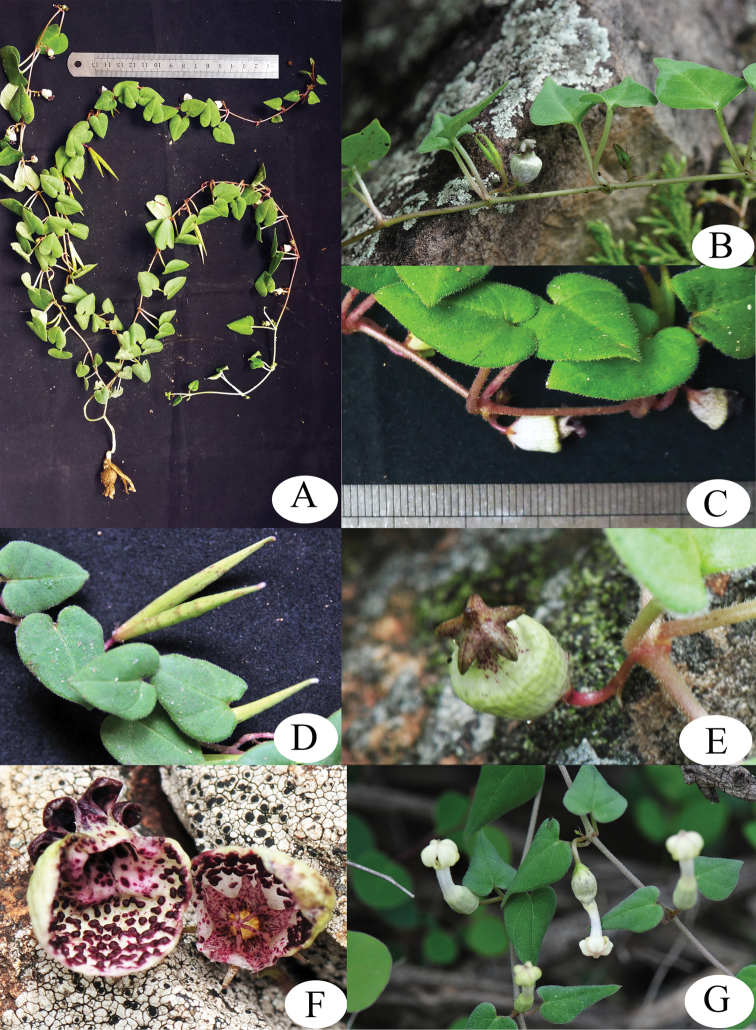
Morphological comparisons of *C. jinshaensis* and closely related species: **A–F***C. jinshaensis***A** plant **B, C** leaves and flowers **D** immature follicles **E** front view of flower showing pentagonal canopy **F** dissected corolla tube showing interior of basal corolla tube and corona **G** leaves and flower of *C. aridicola*. Photographed by Z.K. Wu.

##### Etymology.

*Ceropegia jinshaensis* is named after its type locality, which lies along the Jinsha River.

##### Vernacular name.

Chinese mandarin: jin sha jiang diao deng hua (金沙江吊灯花).

##### Phenology.

This new species was observed flowering from July to August and fruiting from August to October.

##### Distribution and habitat.

*Ceropegia jinshaensis* is currently known from two localities in NW Yunnan and grows on the open stony slope of dry-hot valley along the Jinsha River dominant by *Opuntia monacantha* (Willd.) Haw., *Vitex negundo* L. thicket and with *Hibiscus aridicola* J. Anthony, *Hemerocallis fulva* (L.) L. and *Munronia pinnata* (Wall.) W. Theob.

##### Conservation status.

Only known from two gatherings. The population of type locality is ca. 0.5 km downstream from the crest of Ahai dam; the original habitat could be disturbed or changed by human activities such as road expansion and other building construction. Based on the IUCN criteria 3.1 (IUCN 2012), the conservation status is categorized as Critically Endangered (CR A3c+B2b (iii)).

##### Additional specimens examined

**(paratypes). CHINA**: Yunnan, Yulong County, Fengke, 27°35'35.06"N, 100°26'22.11"E, 1619 m a.s.l., 22 Sep 2015, *J.D. Ya and C. Liu 15CS11213* (paratype KUN!).

## Discussion

The general corolla shape of *C. jinshaensis* is very similar to *C. meleagris* which is restricted to Nepal both in respect of the inflated urceolate corolla tube at the base and the very short lobes. It differs from *C. meleagris* by having shorter leaf blade with cordate base, corolla lobes fused at the top into a pentagonal canopy, glabrous internal surface of entire corolla tube and shorter inner coronal lobes than outer ones. The corolla of *C. jinshaensis* is similar to the Bhutanese species *C. dorjei*. Both of them have the inflated corolla tube at the base and shorter inner lobes than outer lobes of the corona, while the present new species has heart-shaped leaves and much shorter corolla lobes than corolla tube, unlike the corolla lobes in *C. dorjei* which is mostly as long as the corolla tube. The dry-hot valley habitat, non-twining stem and heart-shaped leaves of *C. jinshaensis* remind one of the Chinese endemic *C. aridicola*, which occurs ca. 100 km from the type locality of this new species. However, in *C. aridicola* the apex of the leaf blade is acuminate, the flower is up to 1.5 cm long, the corolla tube is more than twice as long as broad and the flower color is pale green to light pink. A detailed morphological comparison is given in Table 1.

Although the last taxonomic treatment of *Ceropegia* in China recorded 17 species, it is essential to revise the provisional treatment provided in the Flora of China (Li et al. 1995). *C. jinshaensis* is the only new species of *Ceropegia* reported from China since the family was revised two decades ago. This new species presents a corolla with well-inflated corolla tube at base and shorter lobes and this shape is unique among all the known *Ceropegia* species recorded from China. It is crucial to conduct further taxonomic study on this kind of neglected group equipped with up-to-date methods and insight in order to better understand China’s plant diversity before it is too late.

## Supplementary Material

XML Treatment for Ceropegia jinshaensis
